# Significance of K_ATP_ channels, L-type Ca^2+^ channels and CYP450-4A enzymes in oxygen sensing in mouse cremaster muscle arterioles *In vivo*

**DOI:** 10.1186/1472-6793-13-8

**Published:** 2013-05-12

**Authors:** Anh Thuc Ngo, Mads Riemann, Niels-Henrik Holstein-Rathlou, Christian Torp-Pedersen, Lars Jørn Jensen

**Affiliations:** 1 Department of Biomedical Sciences, Faculty of Health and Medical Sciences, The Panum institute, University of Copenhagen, Blegdamsvej 3, Copenhagen N, DK-2200, Denmark; 2 Department of Cardiology, Gentofte Hospital, Copenhagen, Denmark; 3 Department of Veterinary Clinical and Animal Sciences, Faculty of Health and Medical Sciences, University of Copenhagen, Copenhagen, Denmark

**Keywords:** Hypoxic vasodilation, Hyperoxic vasoconstriction, Oxygen sensing, ATP-sensitive K^+^ channels, 20-HETE, L-type Ca^2+^ channels, Prostaglandin, NO-synthase, Skeletal muscle, Arterioles

## Abstract

**Background:**

ATP-sensitive K^+^ channels (K_ATP_ channels), NO, prostaglandins, 20-HETE and L-type Ca^2+^ channels have all been suggested to be involved in oxygen sensing in skeletal muscle arterioles, but the role of the individual mechanisms remain controversial. We aimed to establish the importance of these mechanisms for oxygen sensing in arterioles in an *in vivo* model of metabolically active skeletal muscle. For this purpose we utilized the exteriorized cremaster muscle of anesthetized mice, in which the cremaster muscle was exposed to controlled perturbation of tissue *P*O_2_.

**Results:**

Change from “high” oxygen tension (*P*O_2_ = 153.4 ± 3.4 mmHg) to “low” oxygen tension (*P*O_2_ = 13.8 ± 1.3 mmHg) dilated cremaster muscle arterioles from 11.0 ± 0.4 μm to 32.9 ± 0.9 μm (n = 28, P < 0.05). Glibenclamide (K_ATP_ channel blocker) caused maximal vasoconstriction, and abolished the dilation to low oxygen, whereas the K_ATP_ channel opener cromakalim caused maximal dilation and prevented the constriction to high oxygen. When adding cromakalim on top of glibenclamide or vice versa, the reactivity to oxygen was gradually restored. Inhibition of L-type Ca^2+^ channels using 3 μM nifedipine did not fully block basal tone in the arterioles, but rendered them unresponsive to changes in *P*O_2_. Inhibition of the CYP450-4A enzyme using DDMS blocked vasoconstriction to an increase in *P*O_2_, but had no effect on dilation to low *P*O_2_.

**Conclusions:**

We conclude that: 1) L-type Ca^2+^ channels are central to oxygen sensing, 2) K_ATP_ channels are permissive for the arteriolar response to oxygen, but are not directly involved in the oxygen sensing mechanism and 3) CYP450-4A mediated 20-HETE production is involved in vasoconstriction to high *P*O_2_.

## Background

In the microcirculation, the arterioles regulate tissue blood flow to maintain a close relationship between oxygen supply and demand [1]. Changes in oxygen tension due to changes in the metabolic activity of tissues, particularly in skeletal muscle undergoing vast changes in performance, are believed to be crucial in the regulation of local blood flow. For example, skeletal muscle arterioles dilate/constrict when exposed to a decrease/increase in oxygen tension, thereby controlling the local blood flow to the tissue [1,2].

The mechanisms by which changes in oxygen tension are sensed and converted into downstream signals that lead to vasomotor responses is known as *oxygen sensing*. Because of the importance of oxygen sensing for adjusting skeletal muscle blood flow, several studies have sought to determine its underlying molecular mechanisms. A number of studies have provided evidence which suggests the involvement of ATP-sensitive K^+^ channels (K_ATP_ channels) in the process [3-6], but a similar number of studies from other groups have failed to find evidence to support this notion [7-9]. As an alternative it has been suggested that hypoxia may act on the voltage-dependent L-type Ca^2+^ channels reducing the Ca^2+^ influx into the vascular smooth muscle cells (VSMCs) causing hypoxic vasodilation [9,10]. Nitric oxide (NO) and certain prostaglandins are known to be important endothelium-derived vasodilators and may therefore also play a role in hypoxic vasodilation.

Hypoxic vasodilation may be viewed as a single O_2_-sensitive effector mechanism operating to continuously modify the basal tone of the vessel. However, the operation of dual control mechanisms, i.e. both hypoxic vasodilation and a separate mechanism primarily activated during high oxygen tension to cause vasoconstriction may contribute to an even more elaborate and efficient regulation of local blood flow. The vasoconstrictor 20-hydroxy-eicosatetraenoic acid (20-HETE), which is a ω-hydroxylation product of arachidonic acid produced by the CYP450-4A enzyme system in the presence of molecular oxygen, may be such a mechanism involved in hyperoxic vasoconstriction [11-14].

Many studies have been performed *in vitro*, and have focused on determining the role of a single mechanism for oxygen sensing. However, by examining several proposed mechanisms in an *in vivo* animal model during controlled physiological conditions we are able to study the integrated roles of the different mechanisms for oxygen sensing.

In the present study, we used the exteriorized cremaster muscle in anesthetized male mice. The influence of experimental procedures was minimized by doing only mild surgery under the influence of neurolept anesthesia, which is not known to have any major effects on cardiovascular function [15], and the diameter responses to local tissue oxygen perturbations were observed by intravital microscopy.

Since considerable controversy remains regarding the specific mechanisms responsible for oxygen sensing in skeletal muscle, the main purpose of this study was to address the specific roles of K_ATP_ channels, NO, prostaglandins, 20-HETE and L-type Ca^2+^ channels in this process.

## Methods

### Cremaster muscle preparation

All procedures and protocols were approved by the Danish Animal Care and Use Committee. Male C57BL/6J mice (body weight = 27.6 ± 0.7 g, n = 28; Taconic, DK-8680 Ry, Denmark) were anesthetized by *i.p.* injection with a mixture of droperidol (30.8 mg/kg), midazolam (4.0 mg/kg) and fentanyl (0.2 mg/kg) dissolved in saline (total volume given was 20 ml/kg). This same anesthetic mixture was used to maintain anesthesia, by continuous *i.v.* infusion with the rate of 20 ml/kg/h (syringe pump model 100, serial no. 671, KD Scientific, U.S.A). The mice were tracheotomized to maintain airway patency and the jugular vein was cannulated to allow for infusion of anesthesia and saline, while resting on top of the heated microscope stage. Using gentle dissection, the cremaster muscle with its blood perfusion intact was placed flat on top of a coverslip and fixed by sutures (Prolene 6-0, Ethicon Inc., Somerville, New Jersey, U.S.A.) attached to a silicone bank.

### Superfusion of the cremaster muscle

The Krebs’ solutions (118.4 mM NaCl, 4.8 mM KCl, 2.5 mM CaCl_2_, 1.2 mM MgSO_4_, 25.0 mM NaHCO_3_ and 1.2 mM KH_2_PO_4_, pH range 7.35-7.45) were vigorously bubbled with 95% N_2_/5% CO_2_ or 21% O_2_/74% N_2_/5% CO_2_ gas mixtures to yield low or high oxygen tension Krebs’ solutions respectively. To minimize gas exchange between the superfusate in the supply tubing and the atmospheric air, the length of the tubing was kept at a minimum and thick-walled polyurethane tubing was used (Flexible Tubing 85 Durometer Polyurethane AP01T122PENA, Ark-Plas Products, Inc. Flippin, Arkansas). The superfusate was heated through an inline heater system (Inline heater Model SH-27B, automatic temperature controller TC-324B, Warner Instrument Corporation, Hamden, Connecticut) to a temperature of 34-36°C.

### Vessel diameter measurement

The microcirculation of the cremaster muscle was visualized using a motorized Olympus BX50WI microscope with a fixed stage enabling free positioning of the 4× air- or 20× water-immersion objectives on top of the exteriorized cremaster muscle. The field was viewed on a monitor (Triniton, PWM 1442 QM, Sony, Tokyo, Japan) using a monochrome CCD camera (CCD72S, Dage-MTI Michigan City, IN), and images were recorded on a HDD-recorder (Pioneer DVD recorder DVR-530H) for later off-line analysis. The final magnification (20× objective) of the image was ~700× and the final pixel size was ~0.6 μm. The vessel diameter of the arteriole was measured offline as the external diameter in micrometers (μm).

### Oxygen tension measurement

A fiber-optic oxygen microsensor system (Tapered tip sensor, tip diameter < 50 μm, Microx TX3 Oxygen Meter, PreSens, Regensburg, Germany) was used to measure oxygen tensions in the cremaster muscle microcirculation. The oxygen microsensor consists of a fiber-optic cable with a luminophore at the tip. The luminophore is excited by photons from the oxygen meter. Depending on the presence of molecular oxygen, either photons are reemitted from the luminophore and these are subsequently registered by the oxygen meter, or the energy from the activated luminophore is transferred to the oxygen molecules in the surrounding solution. Thus, the probe does not consume oxygen during the measurements, and consequently, does not by itself affect the oxygen levels within the cremaster muscle tissue. Calibration of the microsensor was performed with a two-point calibration in *oxygen-free water* prepared by diluting 4 g sodium dithionite (Na_2_S_2_O_4_) in 60 ml water in a beaker, and *water vapor-saturated air* prepared by placing wet cotton wool in another beaker. The output of the oxygen sensor is given as the partial oxygen tension in mmHg. All oxygen tension measurements were done with the tip of the oxygen microsensor placed on top of the cremaster muscle in the intersection between the cremaster muscle and the superfusate in close proximity (< 100 μm) to the arterioles under study. The tip, which is the active part of the oxygen microsensor, was always covered by Krebs’ solution and continuous measurements were done throughout the whole experiment with a 1 Hz sampling rate.

### Drugs

To study the mechanisms of oxygen sensing, different drugs were used. Cromakalim (1 and 5 μM), an activator of K_ATP_ channels, glibenclamide (10 and 100 μM), an inhibitor of K_ATP_ channels and nifedipine (3 and 30 μM), a Ca^2+^ antagonist, which inhibits voltage-dependent L-type Ca^2+^ channels, were used to study the role of K_ATP_ channels and L-type Ca^2+^ channels. Dibromo-dodecenyl-methylsulfimide (DDMS, 10 μM), an inhibitor of the cytochrome P450-4A enzyme system (CYP450-4A), was used to study the role of 20-HETE. The role of prostaglandins was studied by using indomethacin (28 μM), an inhibitor of cyclooxygenase. L-NAME (100 μM), an inhibitor of NO-synthases, was used to study the role of NO. Papaverine (100 μM), a smooth muscle cell relaxant, was used to maximally dilate the arterioles to obtain the passive diameter of the arterioles. Cromakalim, glibenclamide and indomethacin were dissolved in dimethyl sulfoxide (DMSO, 0.3%) prior to adding to the Krebs’ solution. The other drugs were dissolved directly in Krebs’ solution. Cromakalim, glibenclamide, indomethacin, L-NAME, nifedipine and papaverine were bought from Sigma-Aldrich, Copenhagen, Denmark, whereas DDMS was bought from Cayman chemical, Michigan, USA.

### Experimental procedures

After preparation of the exteriorized cremaster muscle, the tissue was superfused with low oxygen Krebs’ solution and the preparation was allowed to rest for the next 15-20 minutes to obtain steady state. Subsequently, hyperoxic vasoconstriction and hypoxic vasodilation was induced by superfusing the cremaster muscle with Krebs’ solution containing high or low oxygen tension, respectively. Among several cremaster muscle arterioles (diameter < 40 μm) showing both hyperoxic vasoconstriction and hypoxic vasodilation, one or two arterioles were randomly picked for further study.

The experimental protocol was as follows. During the control period the cremaster muscle was exposed to low oxygen tension followed by high oxygen tension two or three times. Then the cremaster muscle was exposed to low oxygen tension and high oxygen tension in the presence of drugs, and this was repeated at least once. In some experiments two different drugs were added, in other experiments cumulative concentrations of the same drug were added. Afterwards the cremaster muscle was exposed to low oxygen and high oxygen tensions without drugs (wash-out period). At the end of the experiments papaverine was superfused to yield the maximal vessel diameter.

### Data analysis

One or two arterioles were studied per mouse and the outer vessel diameters (D) were measured. ΔD = D_*low oxygen*_ – D_*high oxygen*_, where D_*low oxygen*_ is vessel diameter during low oxygen tension and D_*high oxygen*_ is vessel diameter during high oxygen tension. All results are presented as mean ± SEM. Statistical comparisons were performed by paired two-tailed Student’s *t-*test or one-way ANOVA repeated measures with Holm-Sidak method as post-hoc analysis. P-values < 0.05 were considered as statistically significant.

## Results

**Figure 1:**
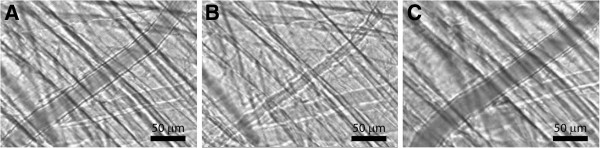
Representative images of a mouse cremaster arteriole during exposure to A) a low oxygen tension, B) a high oxygen tension and C) during application of 100 μM papaverine.

Superfusion with either low or high oxygen Krebs’ solutions had a pronounced effect on the arteriolar diameter and hence on local blood flow to the mouse cremaster muscle. The preparations were stable for 2-3 hours, enough time to study vasomotor responses under different experimental conditions. Images in Figure 1A-C show a representative mouse cremaster muscle arteriole during exposure to low (A), high oxygen tensions (B) and during exposure to a 100 μM papaverine (C). During low oxygen tension (*P*O_2_ = 4.6 mmHg) the external diameter of the arteriole was 32.0 μm. During high oxygen tension (*P*O_2_ = 163.4 mmHg) the arteriole was constricted with external diameter of 9.8 μm. Exposure to 100 μM papaverine (*P*O_2_ = 42.6 mmHg) yielded a maximal external diameter of 35.1 μm. Table 1 summarizes the effects on the vasomotor responses during superfusion with low (*P*O_2_ = 13.8 ± 1.3 mmHg) and high oxygen tensions (*P*O_2_ = 153.4 ± 3.4 mmHg) and 100 μM papaverine (*P*O_2_ = 21.5 ± 1.9 mmHg) from all experiments conducted in this study (n = 28).

**Table 1:** The summarized effects of exposure to low, high oxygen tension and during application of 100 μM papaverine on arteriolar diameter (Mean ± SEM, *p < 0.05 vs. high oxygen and papaverine, †p < 0.05 vs. low oxygen and papaverine, ‡p < 0.05 vs. low oxygen and high oxygen, n = 28)

**n = 28**	**Low oxygen**	**High oxygen**	**100 μM papaverine**
Arteriolar diameter	32.9 ± 0.9 μm *	11.0 ± 0.4 μm †	37.5 ± 1.3 μm ‡
Oxygen tension	13.8 ± 1.3 mmHg	153.4 ± 3.4 mmHg	21.5 ± 1.9 mmHg

### Vehicle control

**Figure 2:**
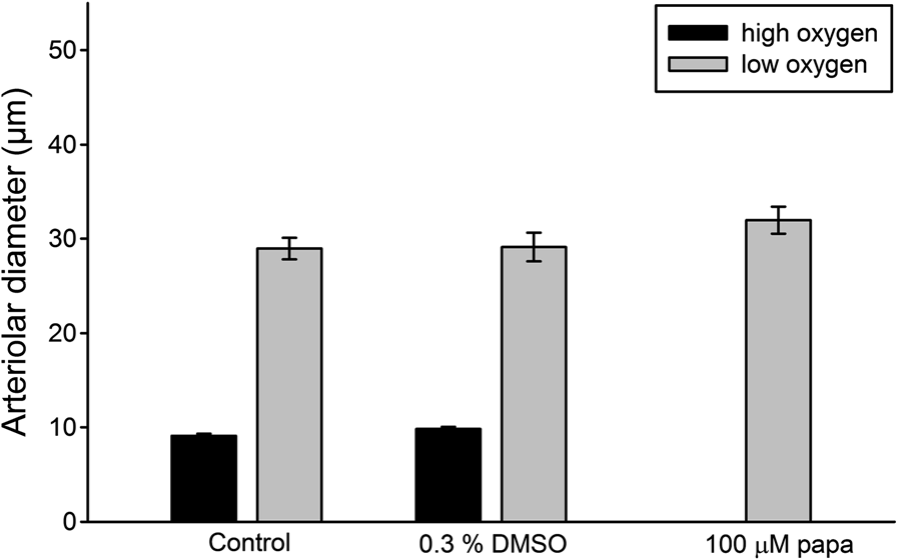
**Effects of DMSO were evaluated.** During the control, high oxygen tension constricted the arterioles, whereas low oxygen tension dilated them. Vessels responded equally in the presence or absence of 0.3% DMSO. There were no statistically significant difference in arteriolar diameter between the control and 0.3% DMSO during low oxygen tension (p = 0.709) and during high oxygen tension (p = 0.188).

DMSO was used to dissolve cromakalim, glibenclamide and indomethacin. DMSO at concentrations up to 0.3% was used in the final Krebs’ solution. To ensure that DMSO did not affect the vascular responses, we tested if there was any difference between the control without DMSO vs. superfusion with Krebs’ solution containing 0.3% DMSO (See Figure 2) (n = 3). During the control, change from high oxygen (*P*O_2_ = 147.6 ± 6.0 mmHg) to low oxygen tension (*P*O_2_ = 16.5 ± 1.7 mmHg) caused a vasodilation (ΔD = 19.9 ± 0.9 μm). In the presence of 0.3% DMSO, change from high oxygen (*P*O_2_ = 145.3 ± 6.5 mmHg) to low oxygen tension (*P*O_2_ = 20.2 ± 2.2 mmHg) caused a similar vasodilation (ΔD = 19.3 ± 1.5 μm) as during the control (p = 0.48, n = 3). Application of 100 μM papaverine yielded the maximal vessel diameter 32.0 ± 1.4 μm.

### Role of K_ATP_ channels

**Figure 3:**
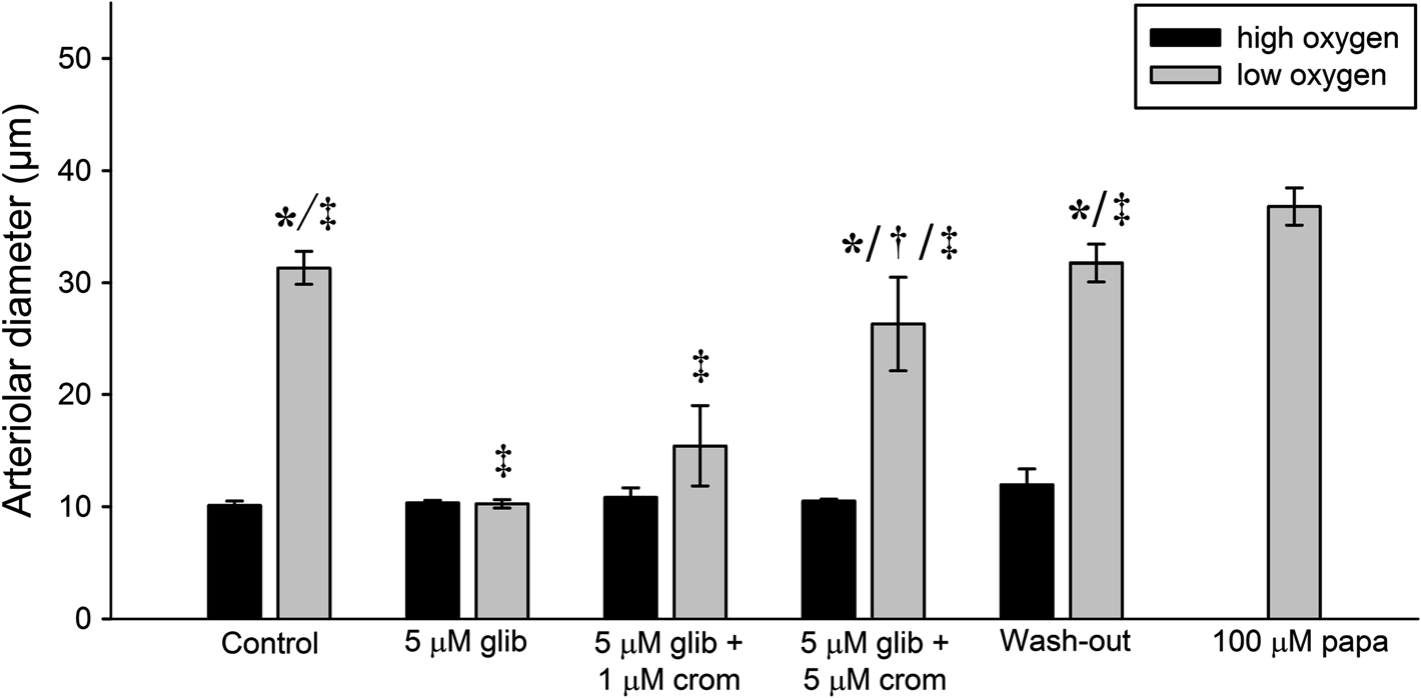
**Effects of consecutive application of 5 μM glibenclamide, 1 μM and 5 μM cromakalim on arteriolar responses during high and low oxygen were evaluated.** (*p < 0.05 vs. high oxygen, †p < 0.05 vs. 5 μM glib and 5 μM glib + 1 μM crom during low oxygen, ‡p < 0.05 vs. papaverine, n = 5).

Figure 3 shows the effects of pharmacological modification of K_ATP_ channel activity using glibenclamide and cromakalim. During the control period, change from high oxygen (*P*O_2_ = 148.4 ± 10.2 mmHg) to low oxygen tension (*P*O_2_ = 12.7 ± 2.6 mmHg) caused vasodilation (ΔD = 21.2 ± 1.9 μm). Subsequent application of 5 μM glibenclamide inhibited vasodilation during low oxygen tension (*P*O_2_ = 9.6 ± 4.6 mmHg). This inhibition could be reversed stepwise by additional application of 1 and 5 μM cromakalim. After a wash-out period lasting ~10 minutes, the vessels regained the same degree of responsiveness to low and high oxygen tensions as during the control period. Application of 100 μM papaverine yielded the maximal vessel diameter 36.8 ± 1.7 μm (n = 5).

**Figure 4:**
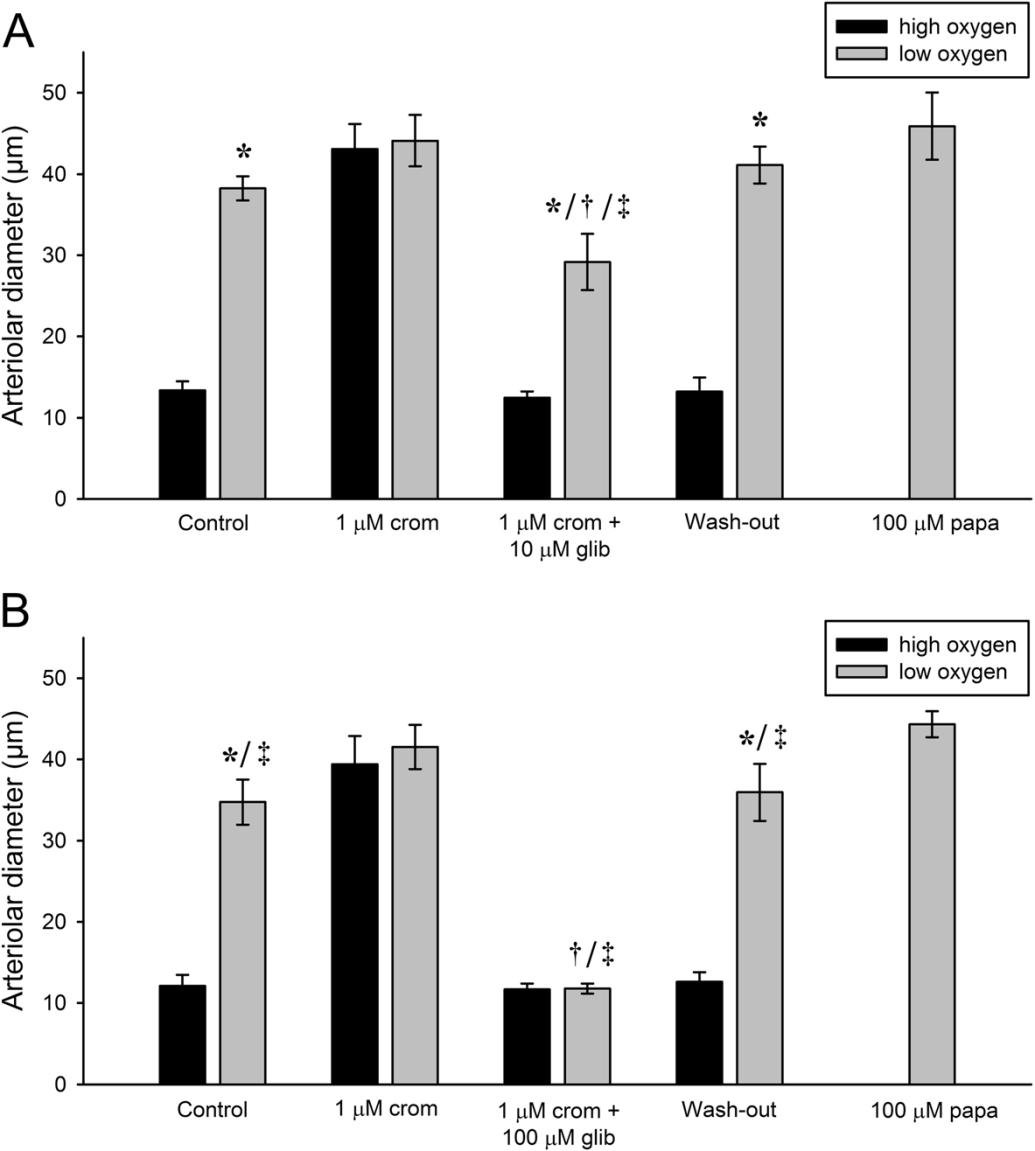
**Effects of consecutive application of 1 μM cromakalim and increasing concentrations of glibenclamide. A**. Effects of consecutive application of 1 μM cromakalim and 10 μM glibenclamide on arteriolar responses during high and low oxygen tensions were evaluated (*p < 0.05 vs. high oxygen, †p < 0.05 vs. control, 1 μM crom and wash-out during low oxygen, ‡p < 0.05 vs. papaverine, n = 5). **B**. Effects of consecutive application of 1 μM cromakalim and 100 μM glibenclamide on arteriolar responses during high and low oxygen tensions were evaluated (*p < 0.05 vs. high oxygen, †p < 0.05 vs. control, 1 μM crom and wash-out during low oxygen, ‡p < 0.05 vs. papaverine, n = 5).

To examine a potential effect of order of application, cromakalim was now applied prior to glibenclamide. In Figure 4A, during the control period a change from high oxygen (*P*O_2_ = 143.5 ± 5.5 mmHg) to low oxygen tension (*P*O_2_ = 10.8 ± 3.1 mmHg) caused vasodilation (ΔD = 24.9 ± 1.2 μm). Subsequent application of 1 μM cromakalim inhibited the vasoconstriction during high oxygen tension. The additional application of 10 μM glibenclamide caused reappearance of the vasoconstriction during high oxygen tension (*P*O_2_ = 153.0 ± 7.1 mmHg), whereas the dilation during low oxygen tension (*P*O_2_ = 7.7 ± 2.2 mmHg) was blunted. In a separate series of experiments (See Figure 4B), 100 μM glibenclamide completely reversed the cromakalim-induced vasodilation. In both series of experiments the vessels regained full responsiveness to changes in oxygen tension following wash-out of the drugs.

### Role of 20-HETE

**Figure 5:**
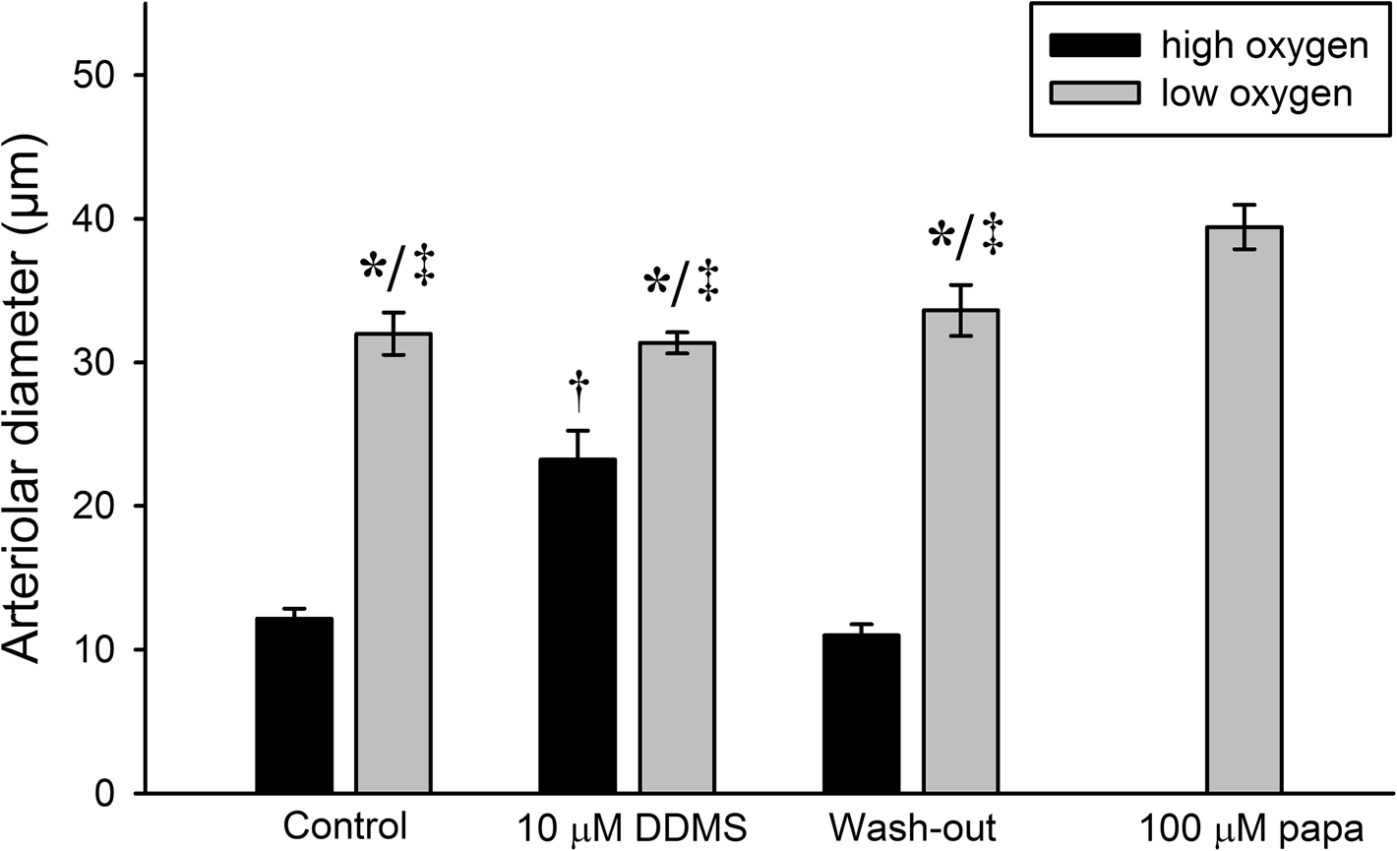
Effects of 10 μM DDMS on the arteriolar responses during high and low oxygen tensions were evaluated (*p < 0.05 vs. high oxygen, †p < 0.05 vs. control high oxygen and wash-out high oxygen, ‡p < 0.05 vs. papaverine, n = 5).

Figure 5 shows the effect of application of DDMS, an inhibitor of 20-HETE production, on vasomotor responses during low and high oxygen tensions (n = 5). During the control period, change from high oxygen (*P*O_2_ = 179.5 ± 6.5 mmHg) to low oxygen tension (*P*O_2_ = 10.3 ± 3.1 mmHg) dilated the arterioles (ΔD = 19.8 ± 1.5 μm). Application of 10 μM DDMS significantly inhibited vasoconstriction during high oxygen (*P*O_2_ = 174.2 ± 3.3 mmHg) (p < 0.05, n = 5), but did not have any effect on arteriolar diameter during low oxygen tension (*P*O_2_ = 8.1 ± 2.8 mmHg). After wash-out, vasomotor responses to changes in oxygen tension were similar to control. Application of 100 μM papaverine yielded the maximal vessel diameter 39.4 ± 1.6 μm.

### Role of L-type Ca^2+^ channels

Both K_ATP_ channels and 20-HETE [4,11] cause vasomotor responses through changes in VSMC membrane potential and voltage-dependent L-type Ca^2+^ channels are therefore a central part of their signaling pathways. Moreover, L-type Ca^2+^ channels have been proposed to be sensitive to changes in the oxygen tension [9,10].

**Figure 6:**
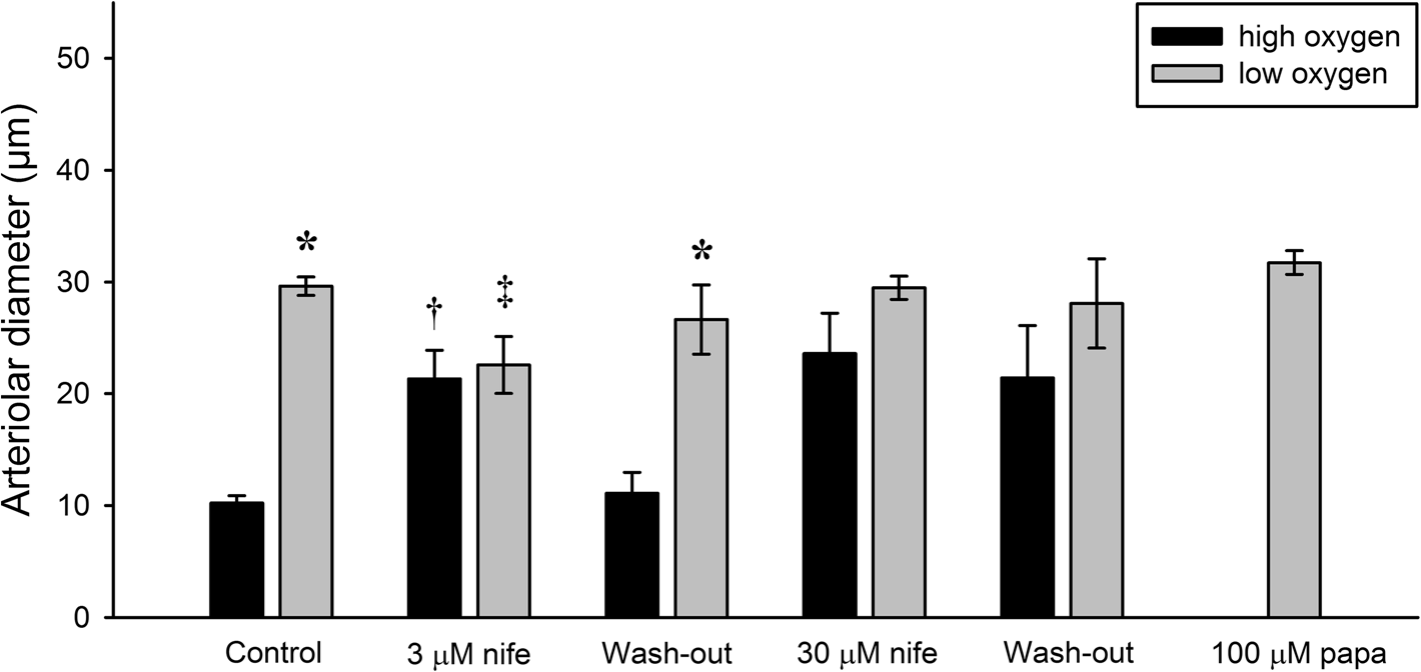
Effects of cumulative stepwise application of 3 μM and 30 μM nifedipine on arteriolar responses were evaluated (*p < 0.05 vs. high oxygen, †p < 0.05 vs. control and washout during high oxygen, ‡p < 0.05 vs. papaverine, n = 5).

Figure 6 shows the effects of blockade of the L-type Ca^2+^ channels by increasing concentrations of nifedipine (3 and 30 μM) on vasomotor responses of the arterioles during both low and high oxygen tensions (n = 5). During the control period, change from high (*P*O_2_ = 167.1 ± 4.3 mmHg) to low oxygen tension (*P*O_2_ = 14.2 ± 3.3 mmHg) caused vasodilation (ΔD = 19.4 ± 1.0 μm). Application of 3 μM nifedipine completely abolished the vasomotor response following the change from high (*P*O_2_ = 159.7 ± 4.6 mmHg) to low oxygen tension (*P*O_2_ = 14.8 ± 1.9 mmHg) in the superfusate. However, the vessels retained a statistically significant basal tone at both low and high oxygen tension. Increasing the concentration of nifedipine to 30 μM abolished basal tone, and a change from high oxygen (*P*O_2_ = 148.9 ± 3.4 mmHg) to low oxygen tension (*P*O_2_ = 9.3 ± 2.4 mmHg) did not cause any significant vasodilation (p = 0.11, n = 5). There were no statistically significant differences between the vessel diameters at high or low oxygen tension at 3 vs. 30 μM nifedipine. Finally, application of 100 μM papaverine yielded a maximal vessel diameter of 31.7 ± 1.0 μm.

### Role of prostaglandins and NO-synthase

**Figure 7:**
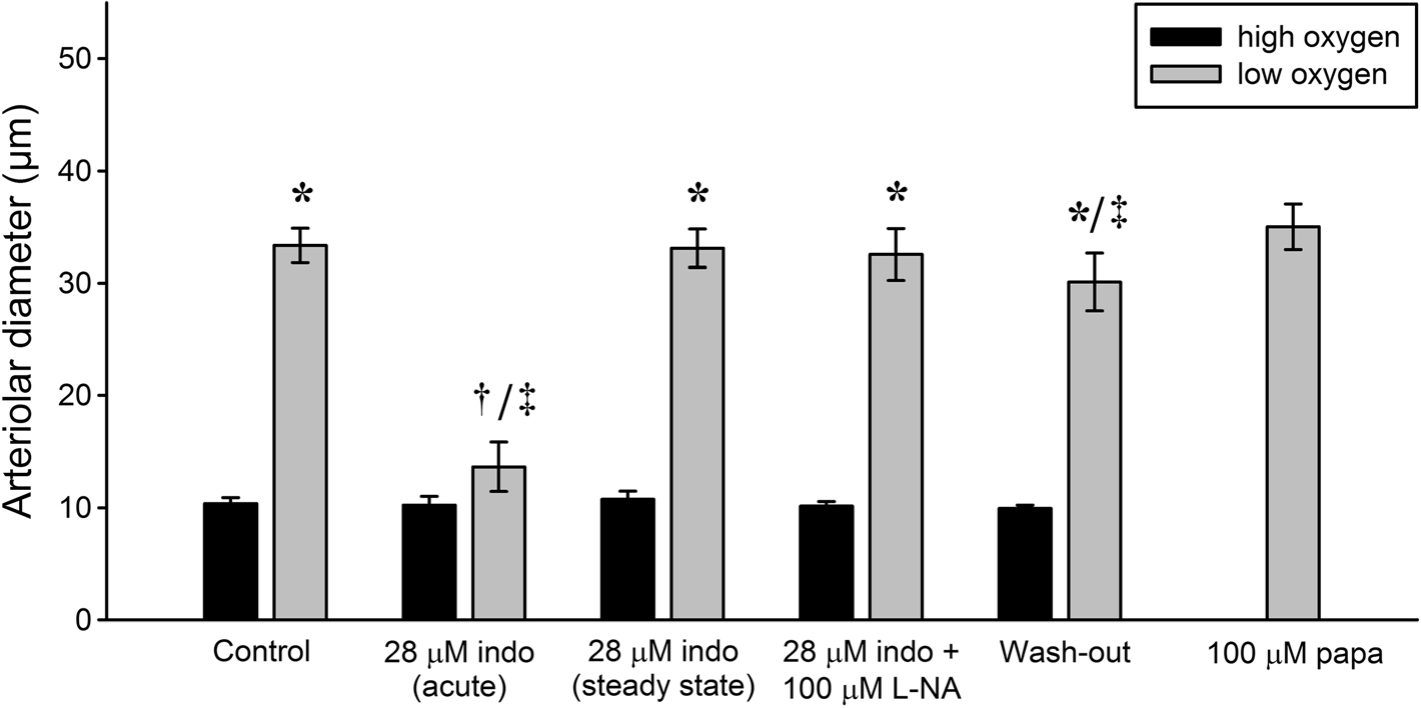
Effects of consecutive application of 28 μM indomethacin and 100 μM L-NAME on arteriolar response during high and low oxygen were evaluated (*p < 0.05 vs. high oxygen, †p < 0.05 vs. control and 28 μm indo (steady state) during low oxygen, ‡p < 0.05 vs. papaverine, n = 5).

Prostaglandins and NO are the two major endothelium-derived vasodilators. Figure 7 shows the effects on vasomotor responses of inhibition of both prostaglandin and NO synthesis, by applying indomethacin followed by L-NAME during both low and high oxygen tensions (n = 5). During the control period, change from high (*P*O_2_ = 148.4 ± 9.9 mmHg) to low oxygen tension (*P*O_2_ = 11.8 ± 5.0 mmHg) caused vasodilation (ΔD = 23.0 ± 1.3 μm). Application of 28 μM indomethacin, a concentration previously shown to effectively block prostaglandin synthesis in hamster cremaster muscle arterioles *in vivo*[16,17], acutely (< 30 sec) inhibited vasodilation during low oxygen tension (*P*O_2_ = 5.9 ± 4.1 mmHg). However, after a period of ~2-3 minutes (steady state) with presence of indomethacin, the vessel regained its responsiveness to low oxygen by dilating again. Additional application of 100 μM L-NAME did not affect vasomotor responses to changes in oxygen tensions.

## Discussion

According to a prominent hypothesis for the mechanism of oxygen sensing in the microcirculation, low oxygen tension opens K_ATP_ channels in VSMCs due, at least in part, to an increase in the ADP/ATP concentration ratio. This leads to efflux of K^+^ and hyperpolarization of the cell, which in turn inhibits the influx of Ca^2+^ through the voltage-gated L-type Ca^2+^ channels leading to smooth muscle relaxation and eventually vasodilation [3,18,19]. In apparent agreement with this notion the present study shows that hypoxic vasodilation was completely inhibited by glibenclamide, and the reactivity could be restored gradually by increasing concentrations of the K_ATP_ channel activator cromakalim (1 μM and 5 μM cromakalim). Likewise cromakalim alone caused maximal vasodilation and a complete loss of hyperoxic vasoconstriction, which was gradually restored by application of 10 μM and 100 μM glibenclamide.

Importantly, however, studies by Jackson have provided evidence against the direct involvement of K_ATP_ channels in hypoxic vasodilation [9]. Jackson showed that low oxygen tension, in a glibenclamide sensitive manner, inhibited norepinephrine-induced contraction in VSMCs from hamster cremaster muscle arterioles. However, the effect of low oxygen tension was neither associated with a change of the whole-cell conductance nor of the membrane potential of the VSMCs [9], which clearly argues against the opening of K^+^ channels. Instead it was suggested that the reported effect of glibenclamide was unspecific, possibly related to a drug-induced membrane depolarization [9].

The results of the present study are therefore difficult to reconcile with a direct role for K_ATP_ channels in oxygen sensing in cremaster muscle arterioles. The most likely explanation of the present results appears to be that the effects of glibenclamide and cromakalim, both when applied alone and in combination, are mediated indirectly through changes in the membrane potential. In cremaster muscle VSMCs glibenclamide applied alone closes K_ATP_ channels, and depolarizes the cell membrane [9]. This will activate the L-type Ca^2+^ channels, and constrict the vessels. We hypothesize that if the membrane depolarization is pronounced, the activation of the L-type channels will be sufficient, even in the presence of hypoxia, to elicit maximal vasoconstriction. The addition of cromakalim antagonizes the effect of glibenclamide and the membrane potential will gradually become more negative as the K_ATP_ channels are reactivated [9]. This will partially deactivate the L-type Ca^2+^ channels due to the steep voltage-dependence of their gating behavior in the physiological range of membrane potentials (–60 to –30 mV), and eventually render them responsive towards the effects of hypoxia. When cromakalim is added alone to maximally activate the K_ATP_ channels, the membrane will be hyperpolarized and the L-type Ca^2+^ channels closed. The vessel will therefore be close to maximally dilated, and once more irresponsive to changes in oxygen levels.

These apparently competitive actions of glibenclamide and cromakalim on the K_ATP_ channels are in line with previous *ex vivo* as well as *in vivo* observations. In isolated porcine coronary artery, the concentration-response curve of glibenclamide was right-shifted in the presence of cromakalim demonstrating a competitive action of these drugs on K_ATP_ channels [20]. In an elaborate *in vivo* study in rats, Gardiner et al showed that the hindquarter skeletal muscle blood flow was increased significantly by levcromakalim, and that this was reversibly antagonized by glibenclamide, demonstrating that glibenclamide and a cromakalim analogue have reversible and competitive actions on local blood flow in rat skeletal muscle *in vivo*. Similar effects were obtained in the mesenteric and renal circulations [21]. However, in an early pharmacological study glibenclamide (IC_50_ 148 nM) was able to block cromakalim (0.5 μM) induced relaxation of isolated rabbit superior mesenteric artery [22]. As we have used much higher concentrations of these drugs due to the fact that it was an *in vivo* study, we speculate that only at sufficiently high concentrations are cromakalim able to compete with the actions of glibenclamide on K_ATP_ channels. We find it unlikely that the observed effects represent unspecific effects due to the high concentrations of the respective drugs. However, we cannot rule out this possibility, and it is clearly a limitation of the present *in vivo* study.

The results of the glibenclamide/cromakalim experiments show that oxygen sensing depends critically on the level of the membrane potential, and this suggest that voltage gated L-type Ca^2+^ channels could play a central role in the mechanism. As is apparent from Figure 6, this was indeed the case. In the presence of medium (3 μM) to high (30 μM) concentrations of nifedipine, there was no significant difference in vessel diameter between high and low oxygen tension. The failure to dilate in response to hypoxia was not due to a complete loss of vessel tone as indicated by the fact that even in the presence of 3 μM nifedipine, the vessels were still able to dilate further when papaverine was added to the superfusate. L-type Ca^2+^ channels are only one of several Ca^2+^ entry mechanisms in VSMCs, and it is therefore not surprising that the vessels are able to retain tone despite the presence of nifedipine. This suggests that the failure to dilate in response to hypoxia was not due to a complete loss of vessel tone, but rather due to the inhibition of the L-type Ca^2+^ channels. When given at a high concentration (30 μM) nifedipine had a more pronounced effect on vascular tone, and this effect was irreversible within the time limit of the washout period. The fact that changes in Ca^2+^ influx through the L-type channels appears to be involved in oxygen sensing does not indicate that the change in influx is mediated by changes in the membrane potential. Previous studies have suggested that hypoxia could induce vasodilation by acting directly on the voltage operated L-type Ca^2+^ channels, reducing Ca^2+^ influx [9,10]. Our findings therefore show that L-type Ca^2+^ channels are central for the oxygen sensing mechanism, but they do not allow us to discriminate between a direct action of oxygen on the channels, and an indirect effect mediated through changes in membrane potential.

20-HETE has been proposed to be involved in oxygen sensing by acting as a vasoconstrictor. It is produced in the VSMCs by the CYP450-4A enzyme system with arachidonic acid as a substrate. In the presence of oxygen, CYP450-4A oxidizes arachidonic acid to 20-HETE. The *K*_*m*_ for oxygen is approximately 55 μM at 37°C [12]. This value corresponds to an oxygen tension of around 40 mmHg, which is above the normal values in skeletal muscle [2,23-25]. The synthesis of 20-HETE is therefore partially limited by the availability of oxygen, and an increased oxygen tension will lead to an increased production rate for 20-HETE. 20-HETE inhibits large-conductance Ca^2+^-activated K^+^ channels (BK_Ca_ channels) located in the plasma membrane of VSMCs, which leads to depolarization of the cell. This in turn activates voltage-dependent L-type Ca^2+^ channels, increasing influx of Ca^2+^ and causing contraction of the VSMCs and eventually vasoconstriction [11,12,14]. Evidence supporting this includes a patch clamp study on cat cerebral microvessel VSMCs [11] and an intravital microscopy study of rat cremaster muscle arterioles, showing inhibition of hyperoxic vasoconstriction by 17-octadecynoic acid (17-ODYA), an inhibitor of CYP450-4A [12]. In our preparation, inhibition of 20-HETE production using 10 μM DDMS inhibited vasoconstriction to high oxygen tension in a reversible manner, but did not affect vasodilation to low oxygen tension (Figure 5). Previous studies have shown that vessels maintain their reactivity to vasoconstrictors (norepinephrine) following addition of DDMS [26], and in the present study the vessels maintained some tone after addition of DDMS. It is therefore unlikely that the lack of response to high oxygen reflects a general failure of the VSMC to contract. The results show that 20-HETE plays a role in vasoconstriction to high oxygen tension in mouse cremaster muscle arterioles, but has no role in hypoxic vasodilation. Since 20-HETE acts as a vasoconstrictor through inhibition of BK_Ca_ channels and/or stimulation of TRPC6 channels [27,28], it is interesting that specific activation of the K_ATP_ channels using cromakalim was able to abolish hyperoxic vasoconstriction (Figure 4A & B). This further strengthens the notion that the effect on oxygen sensing of blocking the K_ATP_ channels is indirect. When the K_ATP_ channels are activated by addition of cromakalim the membrane hyperpolarizes, and we expect that inhibition of the BK_Ca_ channels by 20-HETE will not be able to depolarize the membrane sufficiently to elicit vasoconstriction.

Vasodilator prostaglandins PGE_2_ and PGI_2_ (prostacyclin) and NO released from the vascular endothelium play important roles in regulating vascular tone in the microcirculation [29-33]. Release of prostaglandins and NO has been proposed to occur during low oxygen tension, and these substances could contribute to oxygen sensing [34-38]. Evidence of prostaglandins as effectors of hypoxic vasodilation comes from *in vitro* studies on isolated arterioles, where inhibition of cyclooxygenase using indomethacin inhibited hypoxic vasodilation [37,38]. Moreover, skeletal muscle contraction-induced vasodilation in hamster cremaster muscle arterioles paired with venules was blocked by indomethacin *in vivo*[16].

In our study, inhibition of prostaglandin synthesis by application of 28 μM indomethacin (Figure 7), acutely inhibited vasodilation during low oxygen, but after ~2-3 minutes (steady state) the arterioles had regained their responsiveness, and dilated to low oxygen with a response comparable to the control. The transient reduction in the vessel diameter was probably due to a sudden disruption of a constitutive release of vasodilator prostaglandins causing a transient vasoconstriction. Alternatively, the rate of vasodilation to hypoxia could be severely slowed by the acute lack of prostaglandins. However the vessels quickly regained their responsiveness to low oxygen tension, indicating that prostaglandins are not involved in the steady state mechanism of hypoxic vasodilation. In a previous *in vivo* study, indomethacin inhibited contraction-induced dilation of 3rd order arterioles paired to venules in the hamster cremaster muscle, but had no effect on unpaired 3rd order arterioles. It was therefore suggested that prostaglandins, released from the venules and diffusing to the arterioles, participate in the vasodilator response during muscular contraction [16]. In our study, only unpaired 3rd order arterioles were examined, and in good agreement with the findings of Hammer et al., we did not find evidence for the involvement of prostaglandins in unpaired arterioles in hypoxic vasodilation.

In the present study we tested whether NO was released during low oxygen tension by additional application of 100 μM L-NAME, a concentration which we formerly have shown to effectively abolish bradykinin-induced vasodilation in the mouse cremaster muscle microcirculation *in situ*[6]. In the presence of indomethacin and L-NAME the vessels responded with vasodilation and constriction to low and high oxygen tension as during the control, indicating that NO is not involved in hypoxic vasodilation in mouse cremaster muscle arterioles. This is in good agreement with recent studies from our laboratory, where application of L-NAME did not affect vasomotor responses to low or high oxygen tension [6,39].

The exteriorized cremaster muscle preparation is a well-established *in vivo* model for studying the microcirculation of skeletal muscles. Several studies have investigated the effect of tissue oxygen tension perturbations on vascular responses caused by superfusion of the cremaster muscle with physiological salt solutions equilibrated with gas mixtures with different % O_2_, balanced with N_2_ and 5% CO_2_[40-42]. When the oxygen levels are low the superfusate acts as an “oxygen-sink”, and the tissue oxygen tension is reduced. Conversely, the tissue oxygen tension is increased by elevating the *P*O_2_ of the superfusate, which now behaves as an “oxygen-source” [43,44]. We recently showed that, when the oxygen microsensor tip was placed below the cremaster muscle, a decrease or increase in oxygen tension could be measured that mirrored changes in the superfusate oxygen tension above the tissue [39]. This provides an efficient method for studying the influence of tissue oxygen tensions in the microcirculation of a living animal, and allows changing the oxygen tension locally in the cremaster muscle independently of the oxygen supplied by the vessels. The preparation, on the other hand, does not allow us to determine whether the responses are mediated by oxygen acting directly on the proteins in question, or whether the changes in oxygen levels exert their effect through changes in the production of various metabolites. Nor is it possible to determine the specific cellular location of the signaling pathways, i.e. if they are strictly intravascular or whether they involve the parenchymal cells as well.

## Conclusions

In conclusion, this *in vivo* study supports the notion that K_ATP_ channels are permissive for the response to oxygen by controlling the membrane potential in cremaster muscle VSMCs, whereas the activity of L-type Ca^2+^ channels are central to the oxygen sensing mechanism. As an additional mechanism, 20-HETE mediates hyperoxic vasoconstriction. Our conclusions are highlighted by the fact that blockade of L-type Ca^2+^ channels, which constitute a common pathway for the two mechanisms, inhibited both vasodilation and constriction to low and high oxygen tensions, respectively. In contrast, the lack of effects of inhibiting two major endothelial-derived vasodilator metabolites, prostaglandins and NO, suggests that these pathways are not involved in hypoxic vasodilation in mouse cremaster muscle arterioles *in vivo*.

## Competing interests

The authors declare that they have no competing interests.

## Authors’ contributions

AN conceived and designed the study, carried out all the *in vivo* experiments, analyzed and interpreted the data and drafted and revised the manuscript. MR contributed to the design of the study and helped revising the manuscript. NH contributed to the design of the study, analyzed and interpreted the data as well as helping to draft and revise the manuscript. CT contributed in the design of the study and contributed to drafting and revising of the manuscript. LJ contributed in the conception and design of the study, helped analyzing and interpreting the data and helped to draft and revise the manuscript. All authors read and approved the final manuscript.
